# Toward Development of iMesenchymal Stem Cells for Immunomodulatory Therapy

**DOI:** 10.3389/fimmu.2015.00648

**Published:** 2016-01-06

**Authors:** Samantha F. H. de Witte, Marcella Franquesa, Carla C. Baan, Martin J. Hoogduijn

**Affiliations:** ^1^Nephrology and Transplantation, Department of Internal Medicine, Erasmus Medical Center, Rotterdam, Netherlands

**Keywords:** mesenchymal stem cell, optimization, immunomodulation, cell therapy, secretome

## Abstract

Mesenchymal stem cells (MSC) are under development as an immunomodulatory therapy. The anticipated immunomodulatory effects of MSC are broad, from direct inhibition of lymphocyte proliferation, induction of regulatory T and B cells, to resetting the immune system via a hit-and-run principle. There are endless flavors of MSC. Differences between MSC are originating from donors variation, differences in tissue of origin, the effects of culture conditions, and expansion time. Even standard culture conditions change the properties of MSC dramatically and generate MSC that only remotely resemble their *in vivo* counterparts. Adjustments in culture protocols can further emphasize properties of interest in MSC, thereby generating cells fitted for specific purposes. Culture improved immunomodulatory MSC can be designed to target particular immune disorders. In this review, we describe the observed and the desired immunomodulatory effects of MSC and propose approaches how MSC with optimal immunomodulatory properties can be developed.

## Regulation of Immune Cells by Mesenchymal Stem Cells

Mesenchymal stem cells (MSC) are stromal cells present in connective tissues throughout the body. Their name refers to their ability to differentiate into cells of the mesenchymal lineages, which may be their primary function. Then why would we like to use these cells for immunomodulatory therapy?

Mesenchymal stem cells play an important role in the regulation of the immune system. They are furthermore relatively easy to isolate and can be expanded manifold in culture. Although MSC themselves are not part of the immune system according to the established definitions (1), they interact with all immune cell types. They secrete a large range of anti-inflammatory as well as pro-inflammatory factors, among them cytokines, chemokines, and prostaglandins, which target immune cells and affect their function (2). Among the most highly secreted immune regulatory factors are TGF-β and IL-6, via which MSC direct the induction of regulatory T cells and also Th17 cells (3), the chemo-attractants IL-8, CCL2 (MCP1), CCL8 (MCP2), and prostaglandins E2 and F1 (4). In particular, PGE2 appears to play a central role in the immunoregulatory activity of MSC in several disease models by reprograming macrophages to anti-inflammatory cells and shifting Th1/Th17 responses to Th2 responses (5, 6).

In addition to regulating immune responses via their secretome, MSC express cell surface molecules that undergo interaction with immune cells. For instance, the co-stimulatory and co-inhibitory molecules CD40 and programed death ligand 1 (PD-L1), respectively, are expressed on MSC via which they modulate immune cell activity and proliferation (7, 8). MSC also express immune cell adhesion molecules ICAM-1 and VCAM-1. The expression of these adhesion molecules allows recruitment of activated immune cells to close proximity of MSC, thereby increasing their exposure to anti-inflammatory signals from MSC (9).

In addition to targeting immune cells via soluble and cell membrane signaling pathways, regulation of metabolic pathways takes a central place in the control of immune responses by MSC. MSC are involved in tryptophan metabolism via the expression of indoleamine 2,3-dioxygenase (IDO). IDO depletes tryptophan from the milieu, which results in the inhibition of lymphocyte proliferation (10). Furthermore, MSC catabolize immune-activating ATP to immune-inhibiting adenosine via ecto-5′-nucleotidase (CD73), which is abundantly expressed on MSC (11), thereby regulating the ability of immune cells to build up immune responses. In contrast to human MSC, mouse MSC employ iNOS instead of IDO as a key molecule for immune regulation (12). There are additional differences between species in immune regulatory mechanisms that are involved in the effects of MSC (13). This stresses the fact that there are discrepancies between human and laboratory animal MSC.

## Effects of the Immune Environment on Mesenchymal Stem Cells

Mesenchymal stem cells do not express unique markers and are difficult to identify in tissues. The majority of MSC research is therefore performed on cells in culture. Culture conditions themselves have a large effect on MSC phenotype and function. MSC in culture gain manifolds in size and show shifts in cell surface marker expression (14). Furthermore, culture conditions are supporting a high proliferation rate of MSC, whereas MSC *in vivo* are quiescent, except in situations of repair and growth. Most of what we know about the effect of the immune environment on MSC comes from studies on *in vitro* expanded MSC. It has been demonstrated that the immunomodulatory properties of MSC are strictly regulated by local conditions. Proteins, such as PD-L1, IDO, and IL-6, are strongly upregulated under inflammatory conditions (15). The biological function of this is likely to counterbalance ongoing immune responses and preventing them getting out of hand. In the absence of inflammatory signals, MSC have a mostly supporting function for the immune system (16). MSC support the survival of B cells via yet undetermined factors (17) and prevent T cells from undergoing apoptosis via the secretion of IL-7 (18).

Next to their immune regulatory properties, MSC themselves may become targets of the immune system. While MSC express low levels of HLA and co-stimulatory molecules under standard culture conditions, expression levels are increased by inflammatory stimuli. When allogeneic MSC are used for therapy, this may potentially lead to anti-MSC responses. This has indeed been demonstrated in *in vitro* assays where CD8^+^ T cell responses can be evoked by allogeneic MSC (19). *In vivo* experiments have demonstrated that antibody responses can be detected after repeated injections of allogeneic MSC (20). This indicates that under particular conditions care has to be taken when using MSC of allogeneic origin for therapy.

Mesenchymal stem cells are not only targets of the adaptive immune system but also of the innate immune system. There is clear evidence that autologous culture expanded MSC are lysed by activated NK cells (21, 22). The cause of this may be properties acquired by MSC during culture expansion that trigger cytotoxic responses of NK cells. NK cells may in part be responsible for the short survival time of MSC after intravenous administration (23). They are, however, not solely responsible for the quick disappearance of MSC after infusion as MSC administered to immune deficient mice that lack T, B, and NK cells also have a short survival time (23), indicating other cell types contribute to the clearance of MSC.

Thus, MSC have a broad immune regulatory function, which makes them suitable for immune therapy. The properties of MSC are modified by culture conditions and environmental factors. This can be exploited to generate MSC that have optimal immunoregulatory properties accompanied by a low immunogenicity. Below we discuss which properties of MSC would be desirable for effective and safe therapy and how such MSC can be generated.

## Considerations for the Use of MSC for Immunotherapy

When generating MSC for clinical therapy, there are lots of flavors to choose from. Therapeutic MSC are so far mostly isolated from bone marrow or adipose tissue, but recently umbilical cord has been identified as a rich source of highly proliferative MSC. There have been different approaches to the generation of MSC batches. The approach that is used for several university initiated studies is to generate a small number of low passage MSC doses, whereas the approach that is generally employed by industry-driven studies is to generate large amounts of doses per donor (24). Furthermore, there is the option to cryopreserve MSC before usage, which is the most practical and most widely used option, and there is the option to use MSC directly from culture. Studies have indicated that MSC undergo changes in proliferation rate, adhesion, and bio-distribution after infusion in response to cryopreservation (25, 26), suggesting that cryopreservation may hamper the therapeutic effect of MSC (27).

The route of administration is another aspect that greatly affects the distribution and, thereby, the therapeutic effect of MSC. While MSC express multiple chemokine receptors (28), it is now well established that intravenously infused MSC end up in the lungs due to size restrictions and that there is limited migration of MSC from the lungs to other sites (23, 29). It is therefore questionable whether chemokine receptors play a role in the distribution of MSC after intravenous infusion. This may be different when MSC are administered via different routes, for instance, locally when they may migrate over short distances through tissues to sites of inflammation. There is evidence that endogenous MSC can migrate via the lymphatic system (30) but whether exogenously administered MSC can follow the same path is not known. For the intravenous route of administration, the size of MSC may be determining for the localization of the cells.

The immunogenicity of MSC is also likely to impact the effect of MSC. The first thought may be that non-immunogenic MSC that have a long survival time will have the best immunomodulatory effects and that a rapid disappearance of MSC after administration will impair efficacy. However, the immunogenicity of MSC may also contribute to the immunodulatory effects. We have previously demonstrated that MSC infusion leads to a mild inflammatory response, which is followed by immunosuppression (31). Furthermore, phagocytosis of MSC has been shown to lead to the development of tissue-supportive macrophages (32). There may thus be a balance between the immunogenicity of MSC and their therapeutic effects.

All clinical studies performed with MSC up to date have shown that MSC therapy is safe. There is no evidence for transformation of human MSC and only mild infusion-related adverse effects have been reported (33). The real challenge for MSC immunotherapy is therefore to prove efficacy and one-way to do this is to use MSC with improved immunomodulatory properties. The period of expansion of MSC offers an opportunity to steer MSC to a desired phenotype.

## Improving MSC Therapy

There is growing interest in the development of protocols for the generation of optimized immunomodulatory MSC (iMSC), which can be used to target specific immune disorders. These protocols include modifications of the culture medium, changes in culture conditions, or selection of immunomodulatory subsets of MSC. A frequently used approach to boost the immunomodulatory properties of MSC is the addition of pro-inflammatory cytokines or toll-like receptor (TLR) activators to the MSC culture medium. Modification of culture conditions can include the use of bioreactors and altering oxygen concentrations. Some of these MSC optimizing approaches are discussed below.

## Pro-Inflammatory Stimulation of MSC

### IFNγ

IFNγ is identified as one of the most potent activators of the immunomodulatory properties MSC. IFNγ was first suggested to be a key player in activating the immunosuppressive capacity of MSC in 2006 by Krampera et al. (15). IFNγ-treated MSC demonstrated improved capability of suppressing the proliferation of CD4^+^ and CD8^+^ T lymphocytes and NK cells, in a contact-independent manner. Studies have shown that IFNγ induces IDO expression in MSC (10), and increases PGE2, hepatocyte growth factor (HGF), and TGFβ production (34, 35). PGE2 inhibits T-cell proliferation, induces IL-10 production, and reduces TNFα, IL-12, IL-1β, and IL-8 expression in macrophages (36). The inhibitory co-stimulatory molecule PD-L1 is strongly upregulated in a dose-dependent matter by IFNγ, which leads to the suppression of T-cell proliferation (37). Rafei et al. showed that IFNγ stimulation of MSC also induced CCL2 secretion and that this cytokine has a direct involvement in the inhibition of lymphocyte activation (38).

The efficacy of IFNγ-treated MSC has been investigated in several pre-clinical models. Importance of IFNγ to the immunosuppressive capacity of MSC is highlighted by the fact that MSC from IFNγ*R1*^−/−^ mice are unable to prevent graft versus host disease (GVHD) in contrast to MSC from WT mice (39). Survival of GVHD was 100% when infusing IFNγ-treated MSC compared to the 45% with untreated MSC (40). In addition, IFNγ treatment of MSC enhances their therapeutic activity when they are used in induced colitis models (41).

The immunogenicity of MSC is differentially affected by IFNγ stimulation. Co-stimulatory molecule CD40 and the adhesion molecule CD54 (ICAM-1) are strongly upregulated as well as major histocompatibility complex-I and -II (MHC-I and -II) (9, 34, 37, 38, 42, 43). The upregulation of MHC may result in increased recognition of allogeneic MSC by CD4^+^ and CD8^+^ T lymphocytes, which will result into increased susceptibility of MSC to be lysed by CD8^+^ T lymphocytes. As such, the consequences of IFNγ stimulation on the immunogenicity of MSC could be a reason why in some *in vivo* studies less or no immunosuppressive effects of MSC are seen after IFNγ stimulation, as MSC might already be cleared before they can exert their actions (38). On the contrary, the upregulation of MHC-I reduces lysis of MSC by NK cells (21). Interaction of MHC-I on MSC with receptors on NK cells leads to the activation of inhibitory signals in NK, resulting in decreased release of granules containing perforin and granzymes.

### TNFα

While the effects of IFNγ on MSC have been studied most abundantly, other pro-inflammatory cytokines have potent effects on MSC as well. TNFα has similar, but less pronounced, effects on MSC as IFNγ, such as upregulation of PGE2, IDO, HGF, CD54, and MHC class-I (34, 42, 44). In addition, TNFα upregulates the production of several paracrine factors, including vascular endothelial growth factor (VEGF), fibroblast growth factor 2 (FGF2), and insulin-like growth factor 1 (IGF1) (45). Stimulation with TNFα alone, however, does not influence the immunosuppressive capacity of MSC in a mixed lymphocyte reaction (MLR) (34, 42). Ren et al. showed that IFNγ alone did not suppress T-cell proliferation, but in combination with TNFα, there was a significant suppression (39). Furthermore, IFNγ acted in concert with IL-1α and IL-1β to suppress T cell proliferation. Combinations of these pro-inflammatory factors lead to high expression of CD106 (VCAM-1) and iNOS in MSC and thus the production of NO, resulting in STAT5 phosphorylation in T-cells and suppression of T-cell responses (9, 39, 46). In addition, cocktails of these pro-inflammatory cytokines upregulate several chemokines (CXCL9 and CXCL10), which may bring T-cells, B-cells, and DC into close proximity to the MSC so that MSC can more efficiently exert their immunosuppressive effect (39).

### IL-17A

High expression levels of the IL-17A receptor on the MSC cell surface suggest that IL-17A could be a promising stimulator of MSC immunomodulatory activity. Stimulation with IL-17A increases the proliferation of MSC and has no effects on the MSC morphology or MSC surface marker expressions, including PD-L1 expression. In addition, it has no effect on MHC class I and MHC class II expression; therefore, it appears that MSC maintain their hypo-immunogenicity upon exposure to IL-17 (43). In addition, treatment of MSC with IL-17A enhanced their T-cell suppression capacity to levels observed after IFNγ-treatment (47). Furthermore, IL-17-treated MSC increased IL-6 production and regulatory T cell induction from activated CD3^+^ T lymphocytes, downregulated CD25 expression by CD4^+^ T lymphocytes, and inhibited Th1 cytokine secretion (IFNγ, TNFα, IL-2, and IL-10) (43). These results suggest that IL-17 stimulation improves the therapeutic properties of MSC.

### Activation of Toll-Like Receptors

Activation of TLR on MSC has been touched upon in a number of studies. MSC express high levels of TLR3 and TLR4 and their activation changes the phenotype and immunomodulatory properties of MSC. There are data that indicate that TLR activation stimulates the expression of immunosuppressive factors (IDO and PGE2) by MSC and enhances the inhibitory effects of MSC on immune cells (48). Other studies demonstrate that stimulation of TLR4 on MSC induces a more pro-inflammatory phenotype of MSC with high expression of pro-inflammatory factors (CXCL1, IL-6, IL-8, and CCL2) and adhesion molecules (VCAM-1 and ICAM-1) and a reduced ability to suppress leukocyte activation (49–51). By contrast, upon activation of TLR3 MSC exhibit a milder pro-inflammatory phenotype and show increased leukocyte affinity and maintain their ability to suppress leukocyte activation (49–51). TLR3-stimulated MSC are furthermore better protected against the cytotoxic effects of NK cells (52). It is suggested that TLR activation may represent an effective mechanism to restore immune responses in the case of infection by inhibiting the immunosuppressive effect of MSC (51). Thus, TLR-activated MSC may potentially find applicability as antagonists for regular of enhanced immunosuppressive MSC.

### Other Pro-Inflammatory Factors

Stimulation by pro-inflammatory factors IFNγ, TNFα, IL-17A, and the activation of TLR3 and TLR4 have shown to have a strong effect on the immunomodulatory properties of MSC. The effectiveness of these factors can partially be linked to the high cell surface expression levels of receptors for these factors. Next to the expression of these receptors, MSC also express various other receptors for pro-inflammatory factors on their cell surface, such as for IL-1α, IL-1β, and IL-6 as well as for prostaglandins, suggesting that these factors might as well be effective in modifying the properties of MSC. Studies on the presence of other receptors on MSC will possibly lead to the identification of additional factors that have effects on the properties of MSC. Furthermore, the expression of some of these receptors may be regulated by stimulation with pro-inflammatory factors. This implies that MSC that are encountering a pro-inflammatory environment become more susceptible to other factors, for example, to TGFβ, IL-15, TNFα, and TLR3 and TLR4 activators. The use of cocktails of several pro-inflammatory factors might therefore also be of interest into further improving the immunomodulatory properties of MSC.

## Optimizing Culture Conditions

### Hypoxia

Culture conditions are far from comparable to the *in vivo* conditions. Oxygen concentration is a crucial factor influencing the properties of cells. Standard culture conditions are set at an oxygen concentration of 20%, but in MSC niches in the bone marrow and adipose tissue, depending on the blood flow, this can vary between 3 and 11% (53). Decreasing the oxygen concentration, creating a hypoxic environment, has been shown to increase MSC proliferation and enhance their secretion of VEGF and basic fibroblast growth factor (bFGF) (45, 54). Under hypoxic conditions, MSC are forced to switch to anaerobic metabolism and therefore produce more lactate (44). There is evidence that lactate polarizes macrophages to an anti-inflammatory phenotype (55). Therefore, lactate production may contribute to the immune regulatory function of MSC under hypoxic conditions. Under hypoxia, no signs of MSC toxicity, differences in colony forming unit (CFU) capacity, and other phenotypical changes are observed (44). However, in contrast to what Liu et al. reported, Roemeling et al. saw no changes in the MSC proliferation under hypoxic conditions (44). Nevertheless, during hypoxia, MSC maintained the capacity to induce expression of IDO, PD-L1, and CXCL10 in response to IFNγ and TNF-α (44). In addition, while T cell proliferation is impaired under hypoxic conditions, T-cell inhibition by MSC under hypoxia is maintained and therefore relatively more prominent than under normoxia (44). The absence of oxygen thus appears to enhance the immunomodulatory properties of MSC in biological systems.

### Bioreactors

Next to the conventional static monolayer culture of MSC, bioreactors are a novel way of expanding MSC. In bioreactors, culture conditions can be accurately controlled and strains and stresses can be exerted on the MSC due to the media flow (56, 57). In these bioreactors, MSC can be seeded on polymer-specific scaffolds and the dynamic media flow (due to the two- or three-dimensional rotation of the bioreactor) applies a mechanical stimulus to the MSC. It was seen that the proliferation and the distribution of the MSC in the scaffolds increased when the MSC were in the bioreactor. No changes in their immunogenicity were observed as MHC-I and -II expression was unaffected (56, 57). Mechanical vibrations are used to stimulate the differentiation of MSC into various lineages. In addition to affecting the differentiation potential of MSC, vibrations influenced the immunomodulatory properties of MSC by increasing expression of IL-1, TGFβ1, and TNFα (58). It has yet to be investigated what the effects of culturing in bioreactors are on the immunomodulatory properties of MSC.

### Scaffolds

Scaffolds provide a surface for MSC in culture and help to retain and deliver MSC. Scaffolds made from type-I collagen, hydrogels, sponges, and membranes provide different microenvironments for MSC (59). MHC class-I and -II expression increases gradually on MSC seeded on these scaffolds, with the least increase seen in hydrogel seeded MSC. Presence of IFNγ further increases the expression of MHC class-II. In a one-way MLR, MSC seeded in a hydrogel evoked a low lymphocyte response, whereas MSC in a sponge and membrane enhanced lymphocyte proliferation, compared to MSC which were not on any scaffold (59). The use of these different scaffolds, made from the same material, has a differential effect on MSC’s immunological properties.

### Engineering

Currently, innovative techniques for engineering MSC are under development. Of interest for the development of iMSC is the use of intracellular agent-loaded microparticles (60). The technique enables the loading of MSC with particles containing agents controlling the cell’s phenotype and secretome. Agents that improve the immunogenicity and immunomodulatory properties of MSC can be incorporated in the particles, which can subsequently target the intrinsic properties of MSC. This could be useful in cases, where in the different phases of cell therapy, changes in the MSC’s phenotype are required. For example, during the initial phase of injection, a specific MSC phenotype with a low immunogenicity might be required, followed by localization of MSC at the site of interest, where the microparticles release their agents and inducing a change in secretome or immunogenicity, which could be necessary for the MSC to fulfill their purpose. This technique will enable the generation of iMSC, which can be customized in a time-dependent manner.

## Subsets of MSC with Enhanced Immunomodulatory Properties

Freshly isolated MSC are a heterogeneous population consisting of various subsets, each with different surface marker expressions, differentiation capacity, gene expression, and secretome as well as immunomodulatory capacity (61). Among these subsets, specific subsets are identified as being immunoprivileged and/or possess superior immunomodulatory capacity. Protocols have been developed for the isolation of these specific MSC subsets. Selection based on surface marker expression results in a more homogeneous and defined MSC subset with potentially enhanced immunomodulatory efficacy. Positive selection on CD271 (low-affinity nerve growth factor receptor, LNGFR) expression generates a CD271^+^ MSC subset with a greater immunosuppressive capacity compared to non-selected MSC (62). A subset of Stro-1^+^ MSC shows enhanced support for human hematopoietic stem cell engraftment and has a greater immunosuppressive capacity, while Stro-1^−^ MSC have a broad distribution after infusion into tissues (63, 64). Other molecules such as CD73 and CD90 are suggested to be important markers to identify MSC subsets with enhanced immunosuppressive capacity (65, 66). Recently, a CD362^+^ (Syndecan-2) subset of MSC has been identified, representing a more homogeneous population of MSC (patent number WO 2013117761 A1). Pre-selected subsets of MSC might be more susceptible to the protocols used to optimize their immunomodulatory properties. Hence, selection of a specific MSC subset may also be another way to generate iMSC.

**Figure 1:**
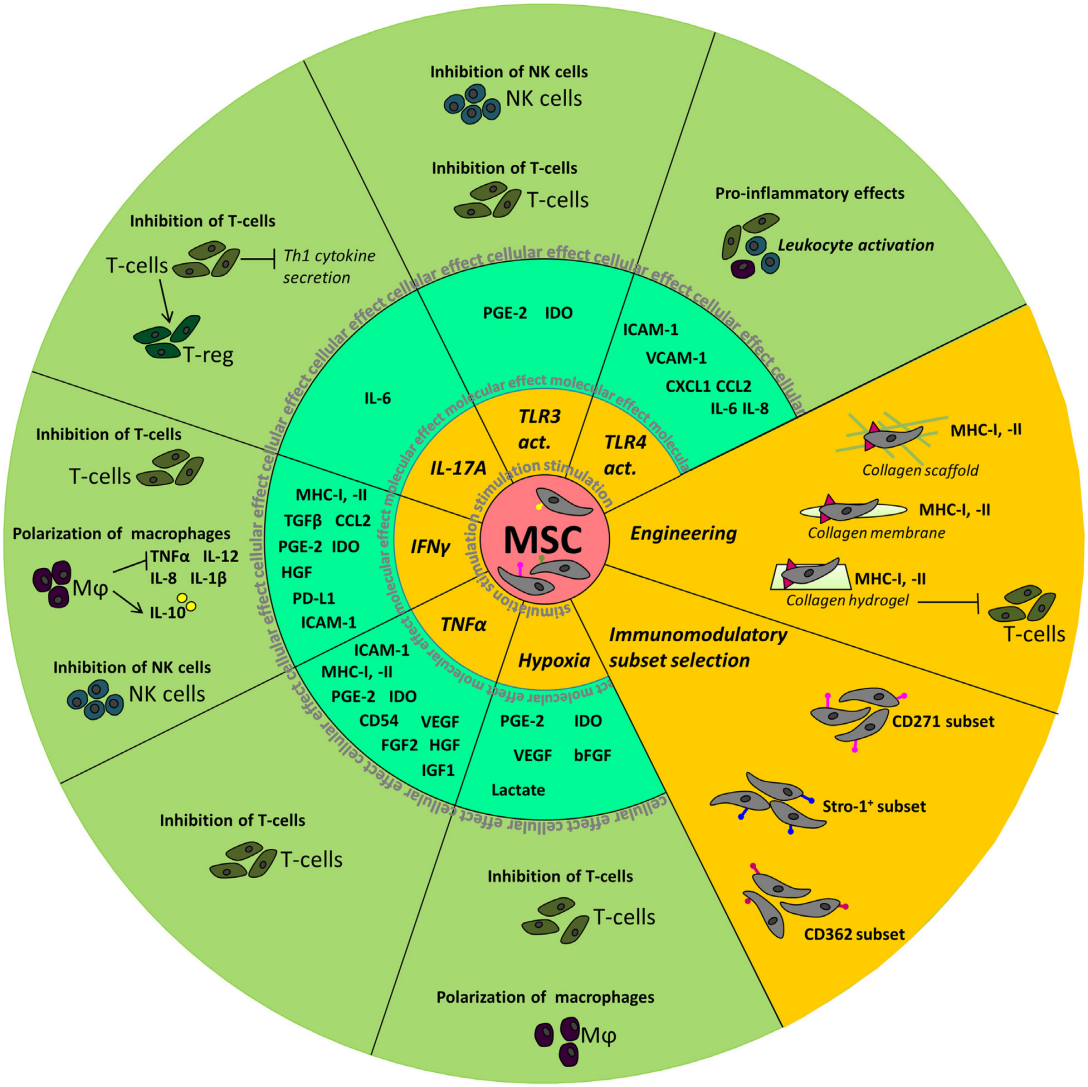
**Summary of the molecular and cellular effects observed after manipulation of MSC**. The inner circle depicts the different stimuli used to manipulate MSC. Molecular effects, such as the upregulation of cytokines and chemokines, seen in MSC are shown in the middle circle. In the outer circle, the effects of stimulated MSC on cellular level are depicted.

A summary of the above described effects of manipulation of MSC is depicted in Figure 1.

## What are the Desired Characteristics of an iMSC?

Immunomodulatory effects of MSC are broad and depending on the disorder, iMSC will be required to possess fitted and specific immunomodulatory properties. Immunological disorders occur in a wide spectrum, with various pathophysiological properties. The design of iMSC is therefore a differential matter where several considerations have to be taken into account. First, immunological diseases can be mediated by one or various immune cells. Specific targeting of T-cell proliferation and activation or manipulation of NK cells, which play a central role in the innate immune response, will be essential in T-cell-mediated diseases or innate immune diseases, respectively. In other conditions, an overall inhibition of an immune response by iMSC might be essential. Second, when infused intravenously MSC are prone to get trapped in the small capillaries of the lungs, due to their size (23, 29). When iMSC are required to have a local effect, manipulation of their size and the presence of tissue-specific chemokine receptors will enable the cells to travel further into the body and to the designated tissue where iMSC can exert their immunomodulatory effect. On the other hand, when a systemic effect is desired, iMSC might be required to be in the circulation, or in any arbitrary location where they are trapped, and able to exert a systemic immunomodulatory effect. However, it is known that MSC are short-lived when infused intravenously (23). Within 24 h they die and are cleared from the body. It is possible that MSC become apoptotic after administration. The clearance of apoptotic cells is known to exert immunomodulatory properties (67) and proposed to be used as immunomodulatory therapy in transplantation (68).

Although MSC have short-term effects, long-term effects have also been observed. It is suggested that their short-term effects are mediated by their secretome, whereas their long-term effects are due to interaction and activation of other cell types in a probable hit-and-run way of action. Several studies have reported that the modulation of T-cell responses occur indirectly via MSC-mediated induction of regulatory T-cells (Tregs) (69). These cells are known to play a role in the maintenance of self-tolerance and immune homeostasis. In addition, the induction of regulatory macrophages (Mregs) and regulatory B-cells (Bregs) is also interesting as these cells also play an important role in the regulation of autoimmune and inflammatory diseases. Improved induction of these cells by iMSC may therefore improve the long-term effect of the iMSC therapy when used to treat autoimmune and inflammatory diseases (17, 70, 71). Depending on therapeutic purposes, iMSC might be required to have a short- or long-term effect. Manipulation of the iMSC’s immunogenicity and immunomodulatory properties, including their secretome, may increase their longevity as well as their immunomodulatory effect. As mentioned before, MSC therapy is proven to be safe with no transformations of the MSC with mild side effects observed (33). So therefore, focusing on the manipulation of MSC, thereby acquiring a customized phenotype will be the next step into optimized cell therapy.

## Conclusion

The diversity of immunological disorders demands the generation of differential designs of iMSC. However, currently no clinical trials have been published where iMSC are used as an immunomodulatory therapy. This mainly comes down to the fact that the safety aspects of iMSC are not yet sufficiently explored, and there is still a lot to be done to generate clinical grade iMSC with desired functional characteristics. Furthermore, for many immunological disorders, targets for intervention have yet to be identified, and as a result the desired properties of optimized MSC are not yet known. When more information come in, the current procedure of one MSC therapy for all diseases will be refined and optimized into a customized iMSC treatment, which will be specific and cater to the needs of the targeted disorder.

## Author Contributions

SW and MH: concept and design and writing of the manuscript. MF and CB: concept and design and proofreading of the manuscript.

## Conflict of Interest Statement

The authors declare that the research was conducted in the absence of any commercial or financial relationships that could be construed as a potential conflict of interest.

## References

[1] Hoogduijn MJ Are mesenchymal stromal cells immune cells? Arthritis Res Ther (2015) 17(1):88 10.1186/s13075-015-0596-3 25880839 PMC4377851

[2] Soleymaninejadian E Pramanik K Samadian E. Immunomodulatory properties of mesenchymal stem cells: cytokines and factors. Am J Reprod Immunol (2012) 67(1):1–8. 10.1111/j.1600-0897.2011.01069.x 21951555

[3] Bettelli E Carrier Y Gao W Korn T Strom TB Oukka M Reciprocal developmental pathways for the generation of pathogenic effector TH17 and regulatory T cells. Nature (2006) 441(7090):235–8. 10.1038/nature04753 16648838

[4] Hoogduijn MJ Popp F Verbeek R Masoodi M Nicolaou A Baan C The immunomodulatory properties of mesenchymal stem cells and their use for immunotherapy. Int Immunopharmacol (2010) 10(12):1496–500. 10.1016/j.intimp.2010.06.019 20619384

[5] Nemeth K Leelahavanichkul A Yuen PS Mayer B Parmelee A Doi K Bone marrow stromal cells attenuate sepsis via prostaglandin E(2)-dependent reprogramming of host macrophages to increase their interleukin-10 production. Nat Med (2009) 15(1):42–9. 10.1038/nm.1905 19098906 PMC2706487

[6] Bouffi C Bony C Courties G Jorgensen C Noel D. IL-6-dependent PGE2 secretion by mesenchymal stem cells inhibits local inflammation in experimental arthritis. PLoS One (2010) 5(12):e14247. 10.1371/journal.pone.0014247 21151872 PMC2998425

[7] Augello A Tasso R Negrini SM Amateis A Indiveri F Cancedda R Bone marrow mesenchymal progenitor cells inhibit lymphocyte proliferation by activation of the programmed death 1 pathway. Eur J Immunol (2005) 35(5):1482–90. 10.1002/eji.200425405 15827960

[8] Franco G Guarnotta C Frossi B Piccaluga PP Boveri E Gulino A Bone marrow stroma CD40 expression correlates with inflammatory mast cell infiltration and disease progression in splenic marginal zone lymphoma. Blood (2014) 123(12):1836–49. 10.1182/blood-2013-04-497271 24452203

[9] Ren GW Zhao X Zhang LY Zhang JM L’Huillier A Ling WF Inflammatory cytokine-induced intercellular adhesion molecule-1 and vascular cell adhesion molecule-1 in mesenchymal stem cells are critical for immunosuppression. J Immunol (2010) 184(5):2321–8. 10.4049/jimmunol.0902023 20130212 PMC2881946

[10] Meisel R Zibert A Laryea M Gobel U Daubener W Dilloo D. Human bone marrow stromal cells inhibit allogeneic T-cell responses by indoleamine 2,3-dioxygenase-mediated tryptophan degradation. Blood (2004) 103(12):4619–21. 10.1182/blood-2003-11-3909 15001472

[11] Regateiro FS Cobbold SP Waldmann H. CD73 and adenosine generation in the creation of regulatory microenvironments. Clin Exp Immunol (2013) 171(1):1–7. 10.1111/j.1365-2249.2012.04623.x 23199317 PMC3530089

[12] Ren G Su J Zhang L Zhao X Ling W L’Huillie A Species variation in the mechanisms of mesenchymal stem cell-mediated immunosuppression. Stem Cells (2009) 27(8):1954–62. 10.1002/stem.118 19544427

[13] Romieu-Mourez R Coutu DL Galipeau J. The immune plasticity of mesenchymal stromal cells from mice and men: concordances and discrepancies. Front Biosci (Elite Ed) (2012) 4:824–37. 10.2741/E422 22201917

[14] Braun J Kurtz A Barutcu N Bodo J Thiel A Dong J. Concerted regulation of CD34 and CD105 accompanies mesenchymal stromal cell derivation from human adventitial stromal cell. Stem Cells Dev (2013) 22(5):815–27. 10.1089/scd.2012.0263 23072708

[15] Krampera M Cosmi L Angeli R Pasini A Liotta F Andreini A Role for interferon-gamma in the immunomodulatory activity of human bone marrow mesenchymal stem cells. Stem Cells (2006) 24(2):386–98. 10.1634/stemcells.2005-0008 16123384

[16] Benvenuto F Ferrari S Gerdoni E Gualandi F Frassoni F Pistoia V Human mesenchymal stem cells promote survival of T cells in a quiescent state. Stem Cells (2007) 25(7):1753–60. 10.1634/stemcells.2007-0068 17395776

[17] Franquesa M Mensah FK Huizinga R Strini T Boon L Lombardo E Human adipose tissue-derived mesenchymal stem cells abrogate plasmablast formation and induce regulatory B cells independently of T helper cells. Stem Cells (2015) 33(3):880–91. 10.1002/stem.1881 25376628

[18] Normanton M Alvarenga H Hamerschlak N Ribeiro A Kondo A Rizzo LV Interleukin 7 plays a role in T lymphocyte apoptosis inhibition driven by mesenchymal stem cell without favoring proliferation and cytokines secretion. PLoS One (2014) 9(9):e106673. 10.1371/journal.pone.0106673 25184791 PMC4153662

[19] Roemeling-van Rhijn M Reinders ME Franquesa M Engela AU Korevaar SS Roelofs H Human allogeneic bone marrow and adipose tissue derived mesenchymal stromal cells induce CD8+ cytotoxic T cell reactivity. J Stem Cell Res Ther (2013) 3(Suppl 6):004. 10.4172/2157-7633.S6-004 24729944 PMC3982127

[20] Cho PS Messina DJ Hirsh EL Chi N Goldman SN Lo DP Immunogenicity of umbilical cord tissue derived cells. Blood (2008) 111(1):430–8. 10.1182/blood-2007-03-078774 17909081

[21] Crop MJ Korevaar SS de Kuiper R Ijzermans JN van Besouw NM Baan CC Human mesenchymal stem cells are susceptible to lysis by CD8+ T-cells and NK cells. Cell Transplant (2011) 20:1547–59. 10.3727/096368910X564076 21396164

[22] Spaggiari GM Capobianco A Becchetti S Mingari MC Moretta L. Mesenchymal stem cell-natural killer cell interactions: evidence that activated NK cells are capable of killing MSCs, whereas MSCs can inhibit IL-2-induced NK-cell proliferation. Blood (2006) 107(4):1484–90. 10.1182/blood-2005-07-2775 16239427

[23] Eggenhofer E Benseler V Kroemer A Popp FC Geissler EK Schlitt HJ Mesenchymal stem cells are short-lived and do not migrate beyond the lungs after intravenous infusion. Front Immunol (2012) 3:297. 10.3389/fimmu.2012.00297 23056000 PMC3458305

[24] Galipeau J Krampera M The challenge of defining mesenchymal stromal cell potency assays and their potential use as release criteria. Cytotherapy (2015) 17(2):125–7. 10.1016/j.jcyt.2014.12.008 25593076

[25] Pollock K Sumstad D Kadidlo D McKenna DH Hubel A. Clinical mesenchymal stromal cell products undergo functional changes in response to freezing. Cytotherapy (2015) 17(1):38–45. 10.1016/j.jcyt.2014.06.008 25457275 PMC4274232

[26] Chinnadurai R Garcia MA Sakurai Y Lam WA Kirk AD Galipeau J Actin cytoskeletal disruption following cryopreservation alters the biodistribution of human mesenchymal stromal cells in vivo. Stem Cell Reports (2014) 3(1):60–72. 10.1016/j.stemcr.2014.05.003 25068122 PMC4110775

[27] Moll G Alm JJ Davies LC Von Bahr L Heldring N Stenbeck-Funke L Do cryopreserved mesenchymal stromal cells display impaired immunomodulatory and therapeutic properties? Stem Cells (2014) 32(9):2430–42. 10.1002/stem.1729 24805247 PMC4381870

[28] Sordi V Malosio ML Marchesi F Mercalli A Melzi R Giordano T Bone marrow mesenchymal stem cells express a restricted set of functionally active chemokine receptors capable of promoting migration to pancreatic islets. Blood (2005) 106(2):419–27. 10.1182/blood-2004-09-3507 15784733

[29] Fischer UM Harting MT Jimenez F Monzon-Posadas WO Xue HS Savitz SI Pulmonary passage is a major obstacle for intravenous stem cell delivery: the pulmonary first-pass effect. Stem Cells Dev (2009) 18(5):683–91. 10.1089/scd.2008.0253 19099374 PMC3190292

[30] Gil-Ortega M Garidou L Barreau C Maumus M Breasson L Tavernier G Native adipose stromal cells egress from adipose tissue in vivo: evidence during lymph node activation. Stem Cells (2013) 31(7):1309–20. 10.1002/stem.1375 23533182

[31] Hoogduijn MJ Roemeling-van Rhijn M Engela AU Korevaar SS Mensah FK Franquesa M Mesenchymal stem cells induce an inflammatory response after intravenous infusion. Stem Cells Dev (2013) 22(21):2825–35. 10.1089/scd.2013.0193 23767885

[32] Lu W Fu C Song L Yao Y Zhang X Chen Z Exposure to supernatants of macrophages that phagocytized dead mesenchymal stem cells improves hypoxic cardiomyocytes survival. Int J Cardiol (2013) 165(2):333–40. 10.1016/j.ijcard.2012.03.088 22475845

[33] Lalu MM McIntyre L Pugliese C Fergusson D Winston BW Marshall JC Safety of cell therapy with mesenchymal stromal cells (safecell): a systematic review and meta-analysis of clinical trials. PLoS One (2012) 7(10):e47559. 10.1371/journal.pone.0047559 23133515 PMC3485008

[34] English K Barry FP Field-Corbett CP Mahon BP. IFN-gamma and TNF-alpha differentially regulate immunomodulation by murine mesenchymal stem cells. Immunol Lett (2007) 110(2):91–100. 10.1016/j.imlet.2007.04.001 17507101

[35] Ryan JM Barry F Murphy JM Mahon BP. Interferon-gamma does not break, but promotes the immunosuppressive capacity of adult human mesenchymal stem cells. Clin Exp Immunol (2007) 149(2):353–63. 10.1111/j.1365-2249.2007.03422.x 17521318 PMC1941956

[36] Harris SG Padilla J Koumas L Ray D Phipps RP Prostaglandins as modulators of immunity. Trends Immunol (2002) 23(3):144–50. 10.1016/S1471-4906(01)02154-8 11864843

[37] Sheng H Wang Y Jin Y Zhang Q Zhang Y Wang L A critical role of IFNgamma in priming MSC-mediated suppression of T cell proliferation through up-regulation of B7-H1. Cell Res (2008) 18(8):846–57. 10.1038/cr.2008.80 18607390

[38] Rafei M Birman E Forner K Galipeau J. Allogeneic mesenchymal stem cells for treatment of experimental autoimmune encephalomyelitis. Mol Ther (2009) 17(10):1799–803. 10.1038/mt.2009.157 19602999 PMC2835011

[39] Ren G Zhang L Zhao X Xu G Zhang Y Roberts AI Mesenchymal stem cell-mediated immunosuppression occurs via concerted action of chemokines and nitric oxide. Cell Stem Cell (2008) 2(2):141–50. 10.1016/j.stem.2007.11.014 18371435

[40] Polchert D Sobinsky J Douglas G Kidd M Moadsiri A Reina E IFN-gamma activation of mesenchymal stem cells for treatment and prevention of graft versus host disease. Eur J Immunol (2008) 38(6):1745–55. 10.1002/eji.200738129 18493986 PMC3021120

[41] Duijvestein M Wildenberg ME Welling MM Hennink S Molendijk I van Zuylen VL Pretreatment with interferon-gamma enhances the therapeutic activity of mesenchymal stromal cells in animal models of colitis. Stem Cells (2011) 29(10):1549–58. 10.1002/stem.698 21898680

[42] Prasanna SJ Gopalakrishnan D Shankar SR Vasandan AB. Pro-inflammatory cytokines, IFNgamma and TNFalpha, influence immune properties of human bone marrow and Wharton jelly mesenchymal stem cells differentially. PLoS One (2010) 5(2):e9016. 10.1371/journal.pone.0009016 20126406 PMC2814860

[43] Sivanathan KN Rojas-Canales DM Hope CM Krishnan R Carroll RP Gronthos S Interleukin-17A-induced human mesenchymal stem cells are superior modulators of immunological function. Stem Cells (2015) 33(9):2850–63. 10.1002/stem.2075 26037953

[44] Roemeling-van Rhijn M Mensah FK Korevaar SS Leijs MJ van Osch GJ Ijzermans JN Effects of hypoxia on the immunomodulatory properties of adipose tissue-derived mesenchymal stem cells. Front Immunol (2013) 4:203. 10.3389/fimmu.2013.00203 23882269 PMC3714546

[45] Crisostomo PR Wang Y Markel TA Wang M Lahm T Meldrum DR. Human mesenchymal stem cells stimulated by TNF-alpha, LPS, or hypoxia produce growth factors by an NF kappa B- but not JNK-dependent mechanism. Am J Physiol Cell Physiol (2008) 294(3):C675–82. 10.1152/ajpcell.00437.2007 18234850

[46] Sato K Ozaki K Oh I Meguro A Hatanaka K Nagai T Nitric oxide plays a critical role in suppression of T-cell proliferation by mesenchymal stem cells. Blood (2007) 109(1):228–34. 10.1182/blood-2006-02-002246 16985180

[47] Huang H Kim HJ Chang EJ Lee ZH Hwang SJ Kim HM IL-17 stimulates the proliferation and differentiation of human mesenchymal stem cells: implications for bone remodeling. Cell Death Differ (2009) 16(10):1332–43. 10.1038/cdd.2009.74 19543237

[48] Opitz CA Litzenburger UM Lutz C Lanz TV Tritschler I Koppel A Toll-like receptor engagement enhances the immunosuppressive properties of human bone marrow-derived mesenchymal stem cells by inducing indoleamine-2,3-dioxygenase-1 via interferon-beta and protein kinase R. Stem Cells (2009) 27(4):909–19. 10.1002/stem.7 19353519

[49] Waterman RS Tomchuck SL Henkle SL Betancourt AM. A new mesenchymal stem cell (MSC) paradigm: polarization into a pro-inflammatory MSC1 or an immunosuppressive MSC2 phenotype. PLoS One (2010) 5(4):e10088. 10.1371/journal.pone.0010088 20436665 PMC2859930

[50] Kota DJ DiCarlo B Hetz RA Smith P Cox CS Jr Olson SD. Differential MSC activation leads to distinct mononuclear leukocyte binding mechanisms. Sci Rep (2014) 4:4565. 10.1038/srep04565 24691433 PMC3972508

[51] Liotta F Angeli R Cosmi L Fili L Manuelli C Frosali F Toll-like receptors 3 and 4 are expressed by human bone marrow-derived mesenchymal stem cells and can inhibit their T-cell modulatory activity by impairing notch signaling. Stem Cells (2008) 26(1):279–89. 10.1634/stemcells.2007-0454 17962701

[52] Giuliani M Bennaceur-Griscelli A Nanbakhsh A Oudrhiri N Chouaib S Azzarone B TLR ligands stimulation protects MSC from NK killing. Stem Cells (2014) 32(1):290–300. 10.1002/stem.1563 24123639

[53] Goossens GH Blaak EE. Adipose tissue oxygen tension: implications for chronic metabolic and inflammatory diseases. Curr Opin Clin Nutr Metab Care (2012) 15(6):539–46. 10.1097/MCO.0b013e328358fa87 23037900

[54] Liu L Gao J Yuan Y Chang Q Liao Y Lu F. Hypoxia preconditioned human adipose derived mesenchymal stem cells enhance angiogenic potential via secretion of increased VEGF and bFGF. Cell Biol Int (2013) 37(6):551–60. 10.1002/cbin.10097 23505143

[55] Colegio OR Chu NQ Szabo AL Chu T Rhebergen AM Jairam V Functional polarization of tumour-associated macrophages by tumour-derived lactic acid. Nature (2014) 513(7519):559–63. 10.1038/nature13490 25043024 PMC4301845

[56] Zhang ZY Teoh SH Chong WS Foo TT Chng YC Choolani M A biaxial rotating bioreactor for the culture of fetal mesenchymal stem cells for bone tissue engineering. Biomaterials (2009) 30(14):2694–704. 10.1016/j.biomaterials.2009.01.028 19223070

[57] Zhang ZY Teoh SH Teo EY Khoon Chong MS Shin CW Tien FT A comparison of bioreactors for culture of fetal mesenchymal stem cells for bone tissue engineering. Biomaterials (2010) 31(33):8684–95. 10.1016/j.biomaterials.2010.07.097 20739062

[58] Choi YK Cho H Seo YK Yoon HH Park JK. Stimulation of sub-sonic vibration promotes the differentiation of adipose tissue-derived mesenchymal stem cells into neural cells. Life Sci (2012) 91(9–10):329–37. 10.1016/j.lfs.2012.07.022 22884804

[59] Yuan T Li K Guo L Fan H Zhang X. Modulation of immunological properties of allogeneic mesenchymal stem cells by collagen scaffolds in cartilage tissue engineering. J Biomed Mater Res A (2011) 98(3):332–41. 10.1002/jbm.a.33121 21626664

[60] Ankrum JA Miranda OR Ng KS Sarkar D Xu C Karp JM. Engineering cells with intracellular agent-loaded microparticles to control cell phenotype. Nat Protoc (2014) 9(2):233–45. 10.1038/nprot.2014.002 24407352 PMC4320648

[61] James S Fox J Afsari F Lee J Clough S Knight C Multiparameter analysis of human bone marrow stromal cells identifies distinct immunomodulatory and differentiation-competent subtypes. Stem Cell Reports (2015) 4(6):1004–15. 10.1016/j.stemcr.2015.05.005 26070611 PMC4471830

[62] Kuci S Kuci Z Kreyenberg H Deak E Putsch K Huenecke S CD271 antigen defines a subset of multipotent stromal cells with immunosuppressive and lymphohematopoietic engraftment-promoting properties. Haematologica (2010) 95(4):651–9. 10.3324/haematol.2009.015065 20179086 PMC2857196

[63] Bensidhoum M Chapel A Francois S Demarquay C Mazurier C Fouillard L Homing of in vitro expanded Stro-1- or Stro-1+ human mesenchymal stem cells into the NOD/SCID mouse and their role in supporting human CD34 cell engraftment. Blood (2004) 103(9):3313–9. 10.1182/blood-2003-04-1121 14715641

[64] Nasef A Zhang YZ Mazurier C Bouchet S Bensidhoum M Francois S Selected Stro-1-enriched bone marrow stromal cells display a major suppressive effect on lymphocyte proliferation. Int J Lab Hematol (2009) 31(1):9–19. 10.1111/j.1751-553X.2007.00997.x 19143868

[65] Campioni D Rizzo R Stignani M Melchiorri L Ferrari L Moretti S A decreased positivity for CD90 on human mesenchymal stromal cells (MSCs) is associated with a loss of immunosuppressive activity by MSCs. Cytometry B Clin Cytom (2009) 76(3):225–30. 10.1002/cyto.b.20461 18985728

[66] Beavis PA Stagg J Darcy PK Smyth MJ. CD73: a potent suppressor of antitumor immune responses. Trends Immunol (2012) 33(5):231–7. 10.1016/j.it.2012.02.009 22487321

[67] Poon IK Lucas CD Rossi AG Ravichandran KS. Apoptotic cell clearance: basic biology and therapeutic potential. Nat Rev Immunol (2014) 14(3):166–80. 10.1038/nri3607 24481336 PMC4040260

[68] Saas P Kaminski S Perruche S. Prospects of apoptotic cell-based therapies for transplantation and inflammatory diseases. Immunotherapy (2013) 5(10):1055–73. 10.2217/imt.13.103 24088076

[69] Luz-Crawford P Kurte M Bravo-Alegria J Contreras R Nova-Lamperti E Tejedor G Mesenchymal stem cells generate a CD4+CD25+Foxp3+ regulatory T cell population during the differentiation process of Th1 and Th17 cells. Stem Cell Res Ther (2013) 4(3):65. 10.1186/scrt216 23734780 PMC3706898

[70] Maggini J Mirkin G Bognanni I Holmberg J Piazzon IM Nepomnaschy I Mouse bone marrow-derived mesenchymal stromal cells turn activated macrophages into a regulatory-like profile. PLoS One (2010) 5(2):e9252. 10.1371/journal.pone.0009252 20169081 PMC2821929

[71] Kim J Hematti P. Mesenchymal stem cell-educated macrophages: a novel type of alternatively activated macrophages. Exp Hematol (2009) 37(12):1445–53. 10.1016/j.exphem.2009.09.004 19772890 PMC2783735

